# Rhythmic Firing of Pedunculopontine Tegmental Nucleus Neurons in Monkeys during Eye Movement Task

**DOI:** 10.1371/journal.pone.0128147

**Published:** 2015-06-01

**Authors:** Ken-ichi Okada, Yasushi Kobayashi

**Affiliations:** 1 Osaka University Graduate School of Frontier Biosciences, Suita, Japan; 2 Center for Information and Neural Networks (CiNet), National Institute of Information and Communications Technology, and Osaka University, Osaka, Japan; 3 Osaka University Research Center for Behavioral Economics, Suita, Japan; 4 PRESTO, Japan Science and Technology Agency (JST), Saitama, Japan; McGill University, CANADA

## Abstract

The pedunculopontine tegmental nucleus (PPTN) has been thought to be involved in the control of behavioral state. Projections to the entire thalamus and reciprocal connections with the basal ganglia nuclei suggest a potential role for the PPTN in the control of various rhythmic behaviors, including waking/sleeping and locomotion. Recently, rhythmic activity in the local field potentials was recorded from the PPTN of patients with Parkinson's disease who were treated with levodopa, suggesting that rhythmic firing is a feature of the functioning PPTN and might change with the behaving conditions even within waking. However, it remains unclear whether and how single PPTN neurons exhibit rhythmic firing patterns during various behaving conditions, including executing conditioned eye movement behaviors, seeking reward, or during resting. We previously recorded from PPTN neurons in healthy monkeys during visually guided saccade tasks and reported task-related changes in firing rate, and in this paper, we reanalyzed these data and focused on their firing patterns. A population of PPTN neurons demonstrated a regular firing pattern in that the coefficient of variation of interspike intervals was lower than what would be expected of theoretical random and irregular spike trains. Furthermore, a group of PPTN neurons exhibited a clear periodic single spike firing that changed with the context of the behavioral task. Many of these neurons exhibited a periodic firing pattern during highly active conditions, either the fixation condition during the saccade task or the free-viewing condition during the intertrial interval. We speculate that these task context-related changes in rhythmic firing of PPTN neurons might regulate the monkey's attentional and vigilance state to perform the task.

## Introduction

The rhythmic firing of neurons in the reticular activating system is thought to be essential for the neural encoding process [1]. One of the major nuclei of the reticular activating system is the pedunculopontine tegmental nucleus (PPTN), that has connections with many brain areas and contains various types of neurons (cholinergic, glutamatergic, GABAergic) [2, 3]. Previous studies have revealed the differences between the rostral and caudal parts of the PPTN in neurochemical identity, anatomical connection, and functional role [4, 5]. Studies in slice preparations reported that PPTN neurons are electrically coupled and exhibit a slow tonic repetitive firing pattern in the beta/gamma band range when maximally activated [1, 6]. Classical literature emphasizes that the tonic and rhythmic activity of PPTN neurons play a role in controlling the sleep/wake state and are important for regulating the activation state of the thalamus via their cholinergic projections [7–9]. Single PPTN neurons exhibit high frequency tonic activity during awake and rapid eye movement sleep states, but not during slow-wave sleep [10–12].

Another line of evidence for the rhythmic activity is emerging from recording the population activity of the PPTN in Parkinson’s patients. Treatment with levodopa in patients with Parkinson's disease led to the appearance of alpha band activity (~10 Hz) in the local field potential recorded from the PPTN [13, 14]. Furthermore, alpha frequency oscillation of local field potentials were recorded from the caudal PPTN in Parkinson’s disease patients during walking, and attenuation of alpha activity was associated with gait freezing [15]. These observations suggest that rhythmic firing is a feature of the functioning PPTN and the rhythmicity of PPTN neurons might change with the behaving conditions even within waking. However, the results are limited to recordings of population activity from Parkinson’s disease patients, it remains unclear whether and how single PPTN neurons in healthy animals show regular and rhythmic firing patterns during various behaving conditions, including executing eye movement task, seeking reward, or during the resting state.

We previously recorded single unit activity of PPTN neurons with high temporal fidelity in healthy monkeys during reward conditioned visually guided saccade tasks, and reported task execution- and reward prediction-related tonic increases or decreases in firing rate of PPTN neurons [16, 17]. One possibility is that these neurons also changed their firing regularity and rhythmicity with changing firing rate. While, the other possibility is that another group of neurons showed regular and rhythmic firing pattern throughout the waking epoch.

Here we investigated firing dynamics of single PPTN neurons by reanalyzing data to focus on the regularity of firing during different behavioral conditions such as an active fixation condition during the saccade task (FX-condition) where monkeys predicted given reward size (large or small) by the shape of the fixation target (FT), and a free viewing condition without any task demand during the intertrial interval (ITI) periods (FV-condition). The tendency of PPTN neurons to fire regularly and rhythmically was assessed using the coefficient of variation (CV) of interspike intervals (ISI) and spectral analysis, respectively.

## Materials and Methods

### Subjects

We reanalyzed previously recorded PPTN neuronal activity data obtained from 2 Japanese macaque monkeys (*Macaca fuscata*; monkey 1, male; monkey 2, female) while they executed a reward-conditioned, visually guided saccade task [16, 17]. All experiments were conducted in compliance with the NIH *Guidelines for the Care and Use of Laboratory Animals*. This study was approved by the Committee for Animal Experiments and Ethics at the Osaka University (Permit Number: FBS-13-006). Animals were obtained from Primate Reseach Institute of Kyoto University. Each monkey was kept in individual primate cage at the animal facility in Osaka University, with environmental enrichment (toys) under 12 hour light/dark cycle. They had visual, auditory and olfactory contact with other monkeys. Food and water were available ad libitium at the home cage after each training and recording session. Additionaly, the monkeys were fed a variety of vegetables, fruits, and grains everyday. To assess the monkeys' health, their weight was routinely monitored and expert veterinarian assistance was available on site.

### Surgical procedures

Information of the general methods was published previously [18]. To record neuronal activity and eye movement, a head-holder, chamber for unit recording, and search coil were implanted. All surgery was performed under anesthesia and monitoring vital signs by KO and YK, and all efforts were made to minimize suffering. Monkeys were initially anesthetized with a combination of medetomidine hydrochloride (0.1 mg/kg, i.m.) and ketamine hydrochloride (10 mg/kg, i.m.), and anesthesia was maintained by isoflurane (1~3%). During surgery, isoflurane levels, CO_2_ levels, respiratory rate, EKG, SpO_2_, blood pressure, and rectal temperature were monitored. Antibiotics and analgesics were administered for 1 week after the surgery in order to prevent infection.

### Behavioral task

During experimental session, the monkeys performed the eye movement tasks in a designated primate chair in a dark sound-attenuated room. All aspects of the behavioral experiment were acquired by a real-time data control system (TEMPO; Reflective Computing, St. Louis, MO). Eye position was sampled at spatial resolution of 0.05° and time resolution of 1 ms using search coil method. The visual stimuli were presented on the screen of a 21-inch cathode ray tube monitor at 28 cm from the monkeys.

The monkeys performed a reward-conditioned visually guided saccade task to increase the range of the monkey’s arousal and motivational level to perform the task as described in detail previously [17]. Briefly, the monkeys fixated on the central FT and then made visually guided saccade to peripheral target to obtain juice reward. The reward size (large or small) was indicated by the shape of the FT (triangle, circle, or square). During the experiment, KO and YK monitored limb movements of the monkeys by an infrared camera, and they showed no systematic limb movements.

### Recording procedure

We used tungsten microelectrodes (impedance of 1–6 MΩ, FHC; Bowdoin, ME, USA) for recording the activity of single neurons. Single unit activities were isolated by the shape of action potentials using a template matching algorithm (MSD; Alpha Omega, Nazareth, Israel) and the timing of each spike that matched a spike waveform template were recorded.

The recording chamber was aimed at the PPTN of the monkeys based on MRI (2.2 T; Hitachi, Tokyo, Japan) under general anesthesia with ketamine hydrochloride (10 mg/kg) and pentobarbital (Nembutal; 20 mg/kg) [17]. The electrode were inserted through a guide tube held by a square grid with 1 mm spacing [19] and could be placed reproducibly. Before reaching the PPTN, we monitored auditory responses in the inferior colliculus encountered 3–7 mm before responses in the PPTN, and high-frequency tonic fiber activity in the cerebellar peduncle close to the PPTN.

To comfirm the locations of the recorded neurons, the monkey 2 was deeply anesthetized with pentobarbital (Nembutal; 200 mg/kg) at the conclusion of the recording experiments, and the brains were perfused with 10% formaldehyde. We reconstructed the recording sites for both monkeys from the readings of the micromanipulators and those of the guide grids of the recording chamber referenced to a single marker site [17] and MRI [20].

### Data analysis

Our database consisted of 466 neurons in monkey 1 (bilaterally, 20 tracks), and 171 neurons in monkey 2 (bilaterally, 5 tracks). Only neurons for which there were data from over 40 trials and over 150 spikes were included, which slightly biased our sample toward neurons with high average firing rates. We analyzed the firing pattern of PPTN neurons during 2 different behaving conditions, such as active fixation condition during the saccade task (FX-condition, 1024 ms window before the FT offset) and free viewing condition during ITI (FV-condition, last 1024 ms window of ITI). We calculated the firing rate and CV as the reciprocal of mean and the coefficient of variation during 5 successive ISI window, respectively.

$$CV=\frac{SD(ISI)}{mean(ISI)}$$ (1)

The mean firing rate and CV were calculated by moving the ISI window in 1 ISI step throughout the analyzing epoch across all trials. A high CV was indicative of a random and irregular firing pattern, and a low CV of a rhythmic and regular firing pattern [21, 22].

To quantify the regularity of firing, we performed simulations of Poisson-like random spike trains with a refractory period of 5 ms for a range of mean firing rates between 5 and 100 spikes/s to determine the theoretical relationship between firing rate and CV. We used the results of this simulation as a theoretical borderline (mean—2 SD) to distinguish irregular and regular firing patterns.

The modulation index of the mean firing rate and the CV were calculated for each neuron as:

$$\text{Modulation}\;\text{index}\;\text{of}\;\text{the}\;\text{firing}\;\text{rate}=\frac{\left([\text{firing}\;\text{rate}\;\text{of}\;\text{FX-condition}]-[\text{firing}\;\text{rate}\;\text{of}\;\text{FV-condition}]\right)}{\left([\text{firing}\;\text{rate}\;\text{of}\;\text{FX-condition}]+[\text{firing}\;\text{rate}\;\text{of}\;\text{FV-condition}]\right)}$$ (2)

and

$$\text{Modulation}\;\text{index}\;\text{of}\;\text{the}\;\text{CV}=\frac{\left([\text{CV}\;\text{of}\;\text{FX-condition}]-[\text{CV}\;\text{of}\;\text{FV-condition}]\right)}{\left([\text{CV}\;\text{of}\;\text{FX-condition}]+[\text{CV}\;\text{of}\;\text{FV-condition}]\right)}$$ (3)

A positive value of the modulation index for firing rate and CV indicated an increase in firing rate and CV, respectively, during the FX-condition compared to the FV-condition.

The periodicity and frequency characteristics of PPTN neurons were also quantified using Fourier analyses. The power spectra of the spike trains were calculated using the Fourier transform of the spike trains separately for the FX- and FV-conditions. A neuron was considered to fire periodically if a significant peak was found in the power spectrum between 4 and 150 Hz. For every peak we calculated the threshold from the mean and 4 SD of the power spectrum (200–500 Hz).

We also analyzed the relationship between the changes in firing regularity and changes in firing rate that we previously reported [16, 17]. We classified PPTN neurons as “tonic excitatory” or “tonic suppressive” based on a significant increase or decrease in their firing rate during the post-fixation period (600 ms window after the onset of the initial stimulus) versus their firing rate in the pre-fixation period (600 ms window before the onset of the initial stimulus, *p* < 0.05, Wilcoxon rank-sum test) [16].

We also investigated the relationship between spike durations and the rhythmic firing of neurons, because previous studies reported that the neurotransmitter released by PPTN neurons might be related to their spike durations [23, 24]. Spike duration was measured as the time from the negative phase to after the peak of the spike waveform [18].

## Results

### Firing rate and regularity of PPTN neurons

Previous studies suggested that firing dynamics might play an important role in neuronal computation [25, 26]. We first investigated the firing dynamics of the recorded PPTN neurons by analyzing the basic firing rate and firing regularity (evaluated by CV) during the FX- and FV-conditions. During the FX- condition, the monkeys actively fixated central FT to obtain juice reward. On the other hand, during the FV-condition, the monkeys made large spontaneous saccades at various amplitudes and in various directions [27].

**Fig 1:**
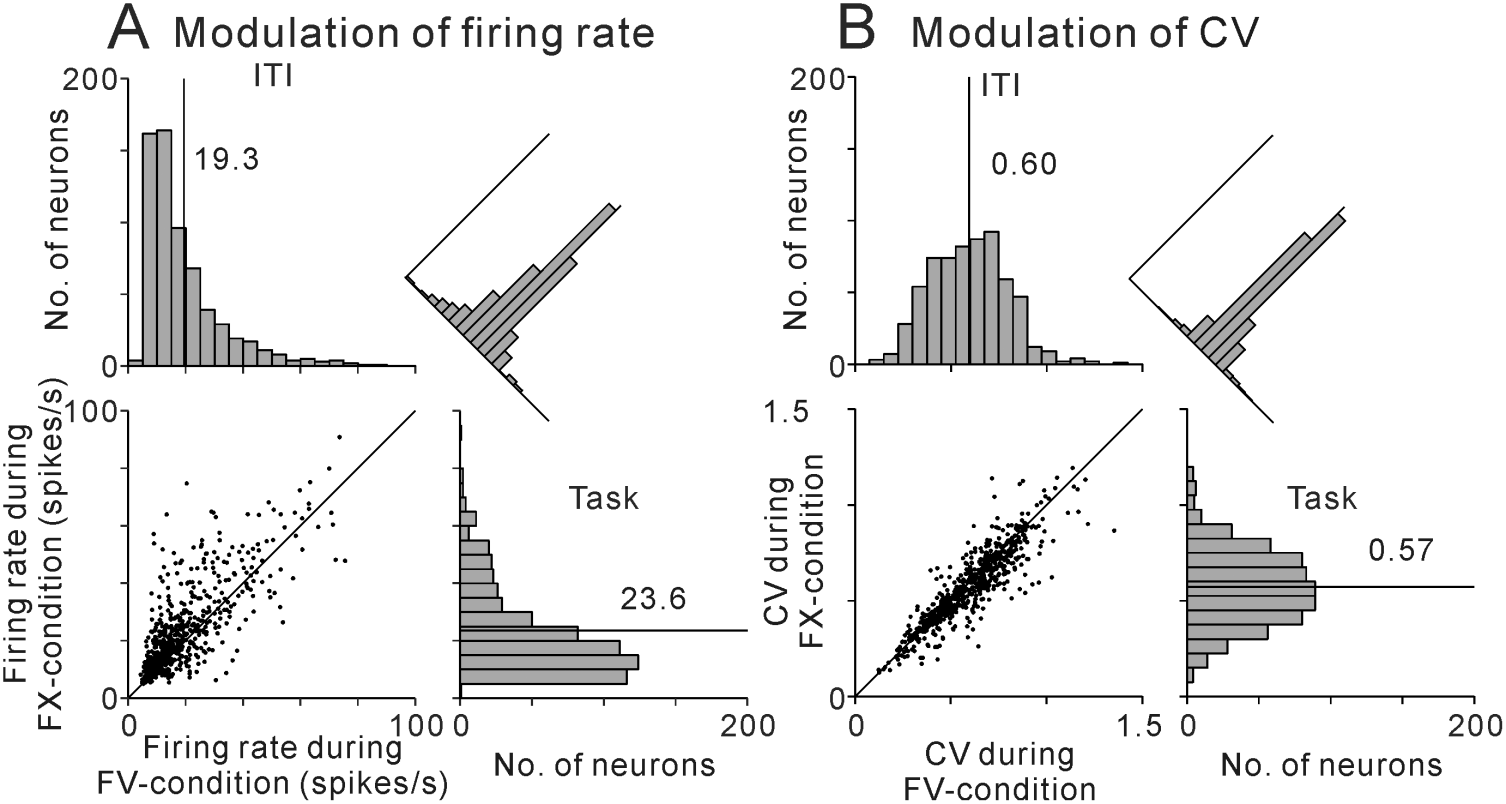
Distributions of firing rate and regularity of PPTN neuron. Summary histograms and scatter plots of the distributions of firing rate (**A**) and regularity (**B**) of PPTN neuron firing during the FX- (*right*) and FV-conditions (*top*).

Fig 1 illustrates the distribution of the average firing rates and CVs during the FX- and FV-conditions. As we reported previously [16], more than half of the PPTN neurons showed significantly higher activity during the FX-condition (57%, N = 364/637, Table 1), resulting in a higher average firing rate during this period (23.6 ± 0.7 spikes/s, mean ± SEM) than during the FV-condition (19.3 ± 0.5 spikes/s, p < 0.001, Wilcoxon signed rank test).

**Table 1:** Summary of task-related changes in firing rate and firing regularity.

	Higher activity during the FV-condition	No change	Higher activity during the FX-condition	Total
Higher regularity during the FV-condition	55	20	48	123
No change	75	66	132	273
Higher regularity during the FX-condition	28	29	184	241
Total	158	115	364	637

Values are number of neurons. Higher regularity was indicated by lower CV.

We then analyzed the firing regularity of PPTN neurons by calculating the CV of the spike trains (Fig 1B). A high CV indicating a variable ISI distribution, a CV = 1 indicating random and irregular firing (variance = mean), and a low CV indicating a regular, rhythmic firing pattern. Almost all PPTN neurons (N = 617, 97%) had a mean CV < 1 during both the FX- (0.57 ± 0.01, mean ± SEM) and the FV-conditions (0.60 ± 0.01). Many neurons showed significantly lower CV during the FX-condition (38%, N = 241/637, Table 1), and as a population, PPTN neurons exhibited a slightly more regular firing pattern during the FX-condition than during the FV-condition (p < 0.05, Wilcoxon signed rank test).

**Fig 2:**
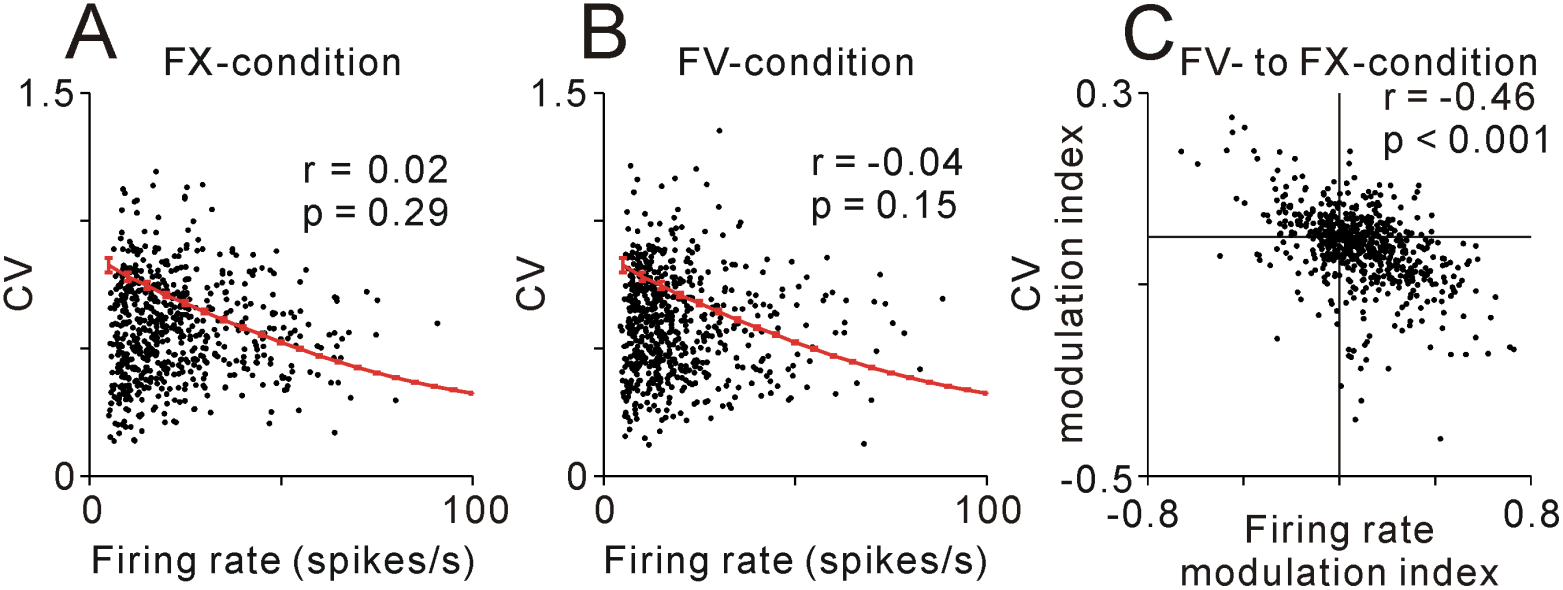
Relationship between firing rate and CV. **(A, B)** Plot of mean CV vs. firing rate of neurons recorded during the FX-condition (**A**, r = 0.02, p = 0.29) or during the FV-condition (**B**, r = -0.04, p = 0.15). Red lines indicate the mean and 1 SD of CV simulated from the random spike trains over a range of mean firing rates between 5 and 100 spikes/s. (**C**) Plot of the modulation of mean CV vs. firing rate as the monkeys’ behavior moved from the FV- to FX-conditions (r = -0.46, p < 0.001), indicating a tendency for increased regularity with increased firing rate.

To further investigate the firing regularity of PPTN neurons, we then analyzed the relationship between the firing rate and the CV (Fig 2A and 2B). The theoretical CV was simulated from the random spike trains for a range of mean firing rates between 5 and 100 spikes/s, and there were a negative relationship between the firing rate and CV (black line). Many PPTN neurons showed a lower CV than that of the theoretical borderline from the simulated random spike trains during both the FX-condition (67%, N = 428, Fig 2A) and the FV-condition (69%, N = 427, Fig 2B). Furthermore, even for neurons with lower firing rate (< 20 spikes/s), part of them had a CV of < 0.5, a value indicating highly regular firing (N = 142/352 and 138/426 for the FX- and FV-conditions, respectively). And thus PPTN neurons showed no significant correlation between the firing rate and CV for the FX-condition (r = 0.02, p = 0.29, Spearman’s rank correlation test) or the FV-condition (r = -0.04, p = 0.15). Overall, the firing characteristics of PPTN neurons were different from the Poisson-like random spike trains in that the CV was lower and not correlated with the firing rate.

Then we analyzed how the relationship between the changes in firing rate and firing regularity of individual neurons for the behavioral task changed from the FV- to FX-conditions. Fig 2C and Table 1 summarize these data during changing task contexts. The positive values of the modulation indices for firing rate and CV indicate an increase in firing rate and CV during the FX-condition compared with the FV-condition. There was a significant negative relationship between the modulation of firing rate and CV (r = -0.46, p < 0.001, Spearman’s rank correlation test). Most of the data in Fig 2C was distributed in the bottom right quadrant, indicating that as the firing rate increased the firing pattern became more regular during the FX-condition compared with those of the FV-condition (N = 184, Table 1). This result suggests that the changes in firing regularity reflected the changes in firing rate within this behavioral context, such that when the firing rate became higher the CV became lower.

Then we analyzed and compared changes in firing dynamics under different conditions that predicted large or small reward during the FX-condition. We previously reported that a group of PPTN neurons showed significant changes in firing rate correlated with the predicted size of given reward and motivation to perform the task [16, 17]. The activity of some neurons increased during the task period and was higher during large reward-predicted trials, while the activity of other neurons decreased during the task period and was lower during large reward-predicted trials [16]. Here we found that the firing regularity of these neurons also changed with the modulation of firing rate, and there was a significant negative relationship between the modulation of firing rate and CV during the FX-condition of large and small reward prediction conditions (r = -0.31, p < 0.001, Spearman’s rank correlation test). Thus, we then used the analysis of covariance to assess the effect of variations in firing rate on the firing regularity observed (1) between the FX- and FV-conditions and (2) between the large and small reward predicted conditions. The slope of the regression line modulated by the FX- and FV-conditions (-0.20) was not significantly different from that modulated by the large and small reward predicted conditions (-0.23, F = 1.09, p = 0.30, analysis of covariance). Thus, the firing regularity of PPTN neurons changed with firing rate, regardless of whether the modulation occurred by the task execution or expectation of reward.

### Rhythmic firing of PPTN neurons

**Fig 3:**
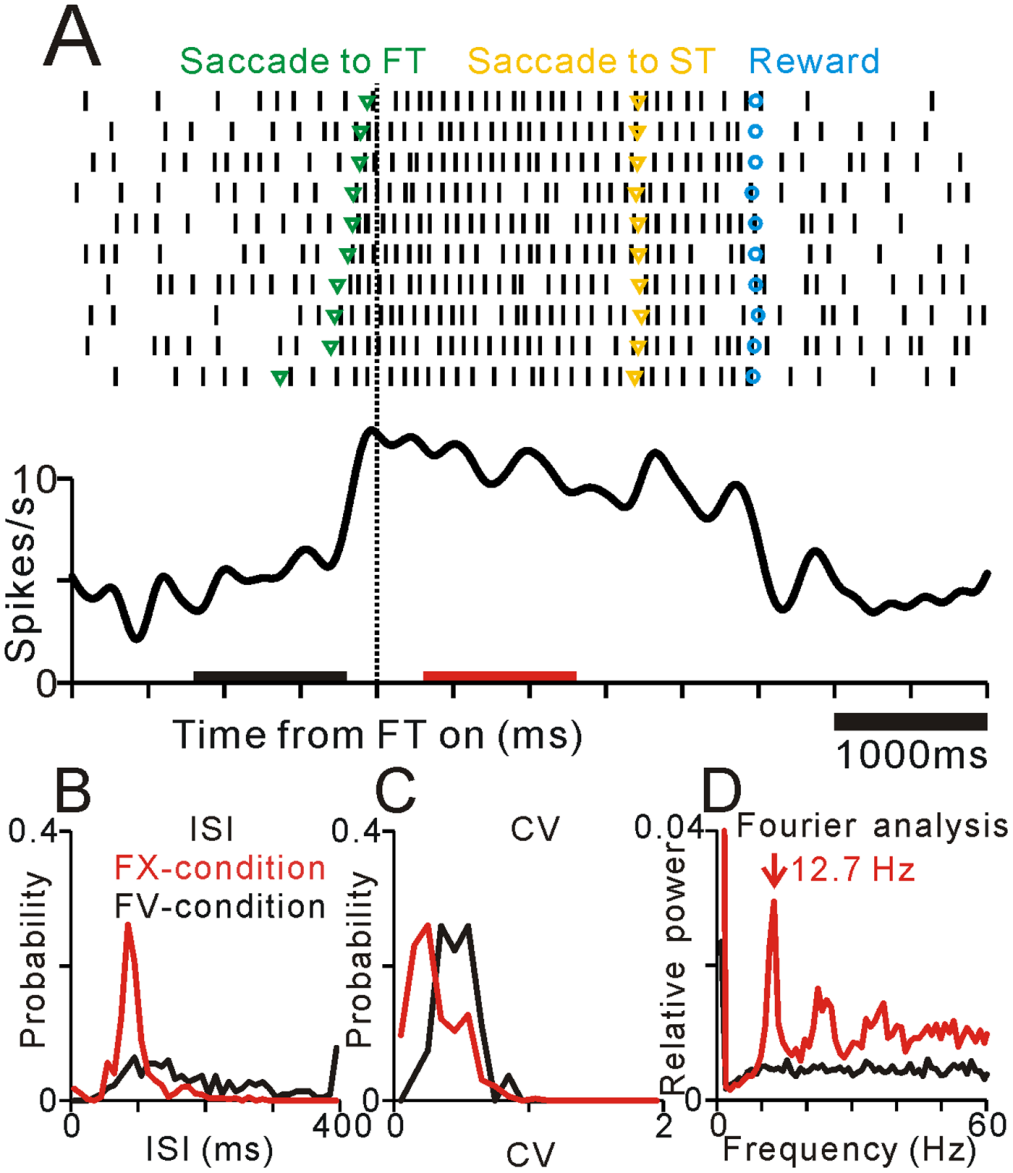
Example of the activity of a typical neuron in the PPTN that exhibited periodic firing during the FX-condition. (**A**) Rastergrams (*top*) and spike density functions (*bottom*). Data are aligned to the appearance of the FT and sorted by relative timing of the centering saccade. This example neuron exhibited periodic firing pattern during the FX-condition. Green, yellow, and blue markers indicate the timing of centering saccades, saccades to target stimulus, and reward delivery, respectively. Black and red bars indicate the FV- and FX-conditions, respectively. (**B-D**) Plots of ISI (**B**), CV (**C**), and power spectrum (**D**) during the FX- (red) and FV-conditions (black).

The rhythmic firing of neurons is thought to be essential for the neural encoding process [1]. Therefore, we investigated the periodicity and frequency characteristics of PPTN neurons by using Fourier analyses. Among our sample of PPTN neurons, 142 of them exhibited a clear periodicity of firing. Fig 3 illustrates an example of a typical PPTN neuron that exhibited a periodic firing pattern only during the FX-condition but not during the FV-condition. On the FV-condition, the neuron exhibited low frequency firing and a rather random firing pattern. Around the time of the FT onset, when the monkey gazed to the center of the screen and fixated, the neuron showed an increase in activity and the firing pattern became more regular and periodic. The ISI histogram of spikes recorded during the FX-condition showed a narrower distribution and sharper peak than that during the FV-condition (Fig 3B), while the CV became lower during the FX-condition (Fig 3C). During this period, the power spectrum indicated a clear low-frequency periodicity (12.7 Hz, Fig 3D). However, this periodic firing pattern faded away during the FV-condition. Rastergrams indicated that the rhythmic firing was composed of repetition of a single spike (Fig 3A), and thus the peak frequency of the power spectrum (12.7 Hz, Fig 3D) corresponded to the mean firing rate (10.3 spikes/s). Thus this representative neuron changed its firing rate and rhythmicity with changing behavioral context that interleaved only a few seconds.

Some neurons exhibited a periodic firing pattern either during the FX-condition (N = 72) or the FV-condition (N = 24), while the others fired periodically throughout the entire behavioral conditions (N = 46). Then we summarized the relationship between the periodic firing patterns and task-related changes in firing rate between the FX- and FV-conditions (Table 2). Generally, PPTN neurons showed periodic firing during highly activated states. Most neurons that showed periodic firing only during the FX-condition exhibited higher activity during this period (81%, N = 58/72). On the other hand, many neurons that showed periodic firing only during the FV-condition exhibited lower activity during the FX-condition and higher tonic activity during the FV-condition (67%, N = 16/24). Thus, both increasing and decreasing types of PPTN neurons showed periodic firing patterns during highly active states.

**Table 2:** Summary of periodic firing patterns and task-related tonic changes in activity.

	Higher activity during the FV-condition	No change	Higher activity during the FX-condition	Total
Periodic firing during the FV-condition	16	4	4	24
Periodic firing during Both conditions	21	7	18	46
Periodic firing during the FX-condition	5	9	58	72

Values are number of neurons.

**Fig 4:**
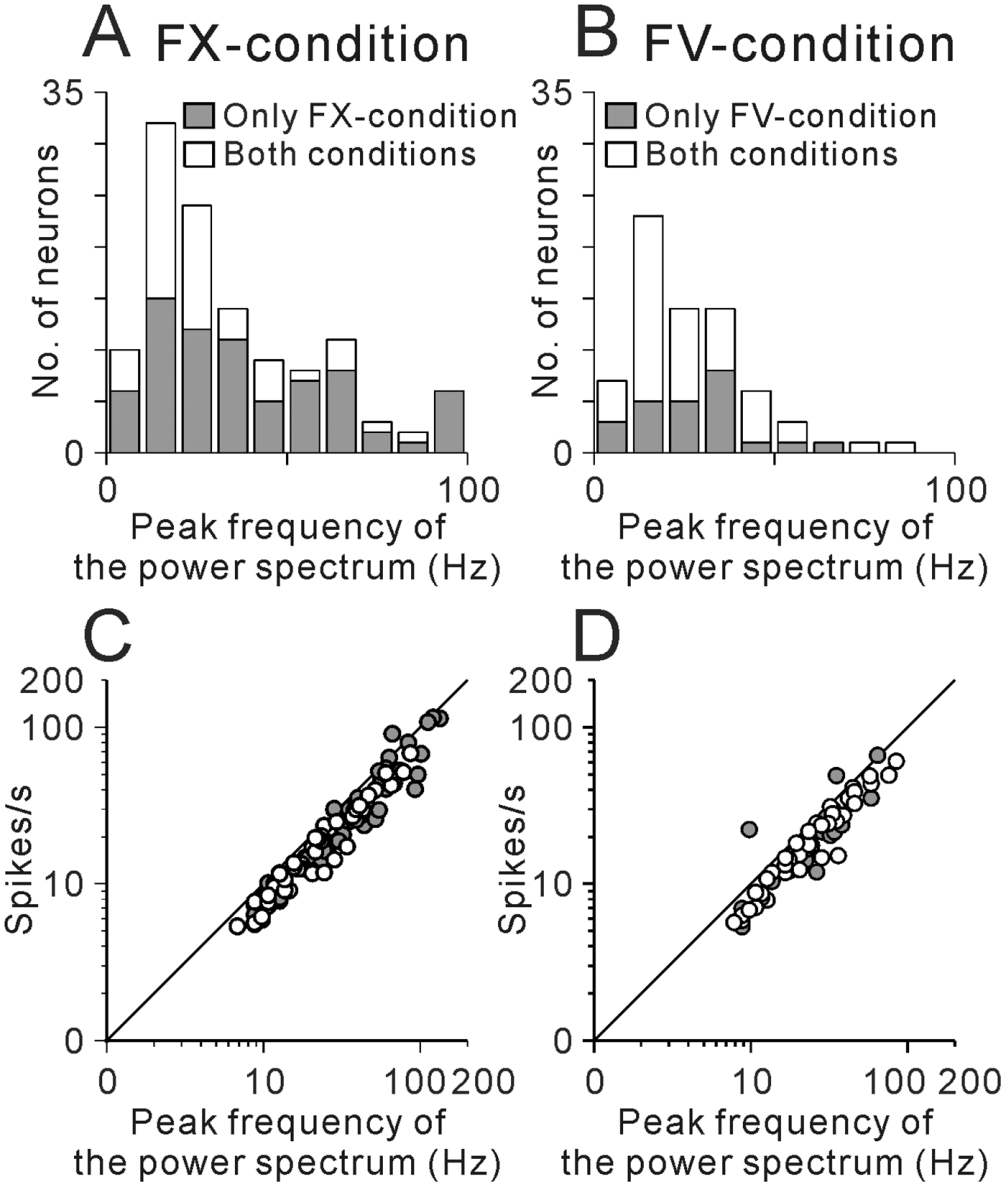
Summary of the frequencies of periodic firing of PPTN neurons. (**A, B**) Summary histograms of the frequency of periodic firing defined by Fourier analysis. Gray bars indicate neurons that exhibited periodic firing only during the FX-condition (**A**) or the FV-condition (**B**), and unfilled bars indicate neurons that exhibited periodic firing throughout the entire behavioral conditions. (**C, D**) Plots of the mean firing rate vs. frequency of periodic firing during the FX- (**C**) or FV-conditions (**D**). Gray circles indicate neurons that exhibited periodic firing only during the FX- (**C**) or FV-conditions (**D**), and open circles indicate neurons that exhibited periodic firing throughout the entire behavioral conditions.

Fig 4 summarizes the distributions of the frequency of periodic firing during the FX- and FV-conditions. More than half of the neurons exhibited periodic firing at low frequency ranging from 5 to 30 Hz (56%, N = 79/142), and the tail of the distribution extended toward the high frequency up to 100 Hz. The average frequency of periodic firing was 35.3 ± 2.4 Hz (mean ± SEM) during the FX-condition and 27.1 ± 2.0 Hz during the FV-condition. For neurons that showed periodic firing during both the FX- and FV-conditions (N = 46), the peak of the power spectrum was slightly changed between the FX- and FV-conditions with the changes in firing rate, but was not significantly different between the two conditions (p = 0.83, Wilcoxon sign rank test). Thus these neurons exhibited almost constant rhythmic firing pattern during recording condition, and possibly throughout the waking condition.

As shown in the analyses of recordings from the example neuron in Fig 3, the rhythmic firing of PPTN neurons was composed of periodically firing single spikes. This feature is further illustrated in Fig 4C and 4D in plots of the mean firing rate and the peak of the power spectrum. If the rhythmic firing is composed of periodically firing single spikes, the mean firing rate corresponds to the peak of the power spectrum. While, if the rhythmic firing is composed of periodic set of burst firing, the peak of the spectrum should be lower than the mean firing rate. The plotted symbols fall along the diagonal lines (Fig 4C and 4D); thus, the frequencies of periodic firing corresponded to the mean firing rate during both the FX- and FV-conditions. The mean firing rates were slightly lower than the peak of the power spectrum, possibly reflecting the fluctuation of firing rate.

### Relationship between the periodic firing pattern and electrophysiological properties of neurons

**Fig 5:**
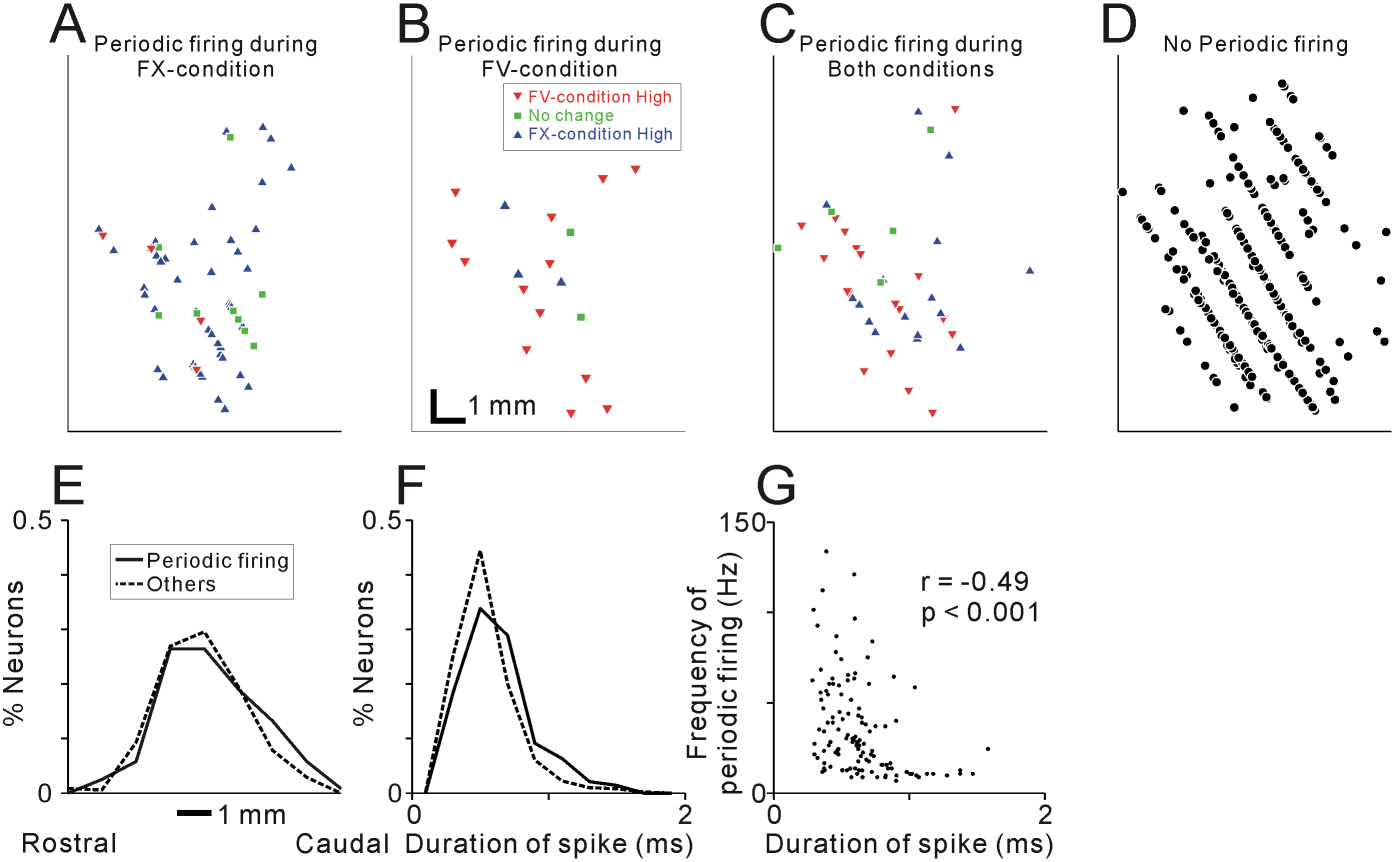
Relationship between the periodic firing pattern with anatomical and physiological properties of neurons. (**A-C**) Distribution of neurons that showed periodic firing during the FX-condition (**A**), FV-condition (**B**), and both conditions (**C**) for monkey 1. Red downward triangles, green squares, and blue upward triangles indicate neurons that showed higher activity during the FV-condition, no significant changes in firing rate, and higher activity during the FX-condition, respectively. (**D**) Distribution of neurons that did not show periodic firing are shown. (**E**) Distribution of the recording location for monkey 1 and (**F**) the duration of spikes for neurons that exhibited periodic firing (solid line) and those that did not (dashed line). (**G**) Relationship between periodic firing frequency and spike duration of recorded neurons. Frequency of periodic firing during the FX-condition plotted against spike duration for the 119 neurons that showed periodic firing during the FX-condition.

In previous studies, the neurotransmitter released by recorded PPTN neurons might be related to their electrophysiological properties, including spiking irregularity, duration, and firing rate [23]. Anatomically, cholinergic and GABAergic neurons are distributed in different parts of the PPTN [28]. Thus, to find a clue of the neurotransmitter profile of periodic firing neurons, we compared the physiological and anatomical properties of the neurons that exhibited periodic firing with those that did not. The mean firing rate of the neurons that fired periodically was not significantly different from those that did not (p = 0.8 for the FX-condition and p = 0.1 for the FV-condition, Wilcoxon rank-sum test). However, there was a significant difference in recording location; although recording location of periodic and non-periodic firing neurons were overlapped (Fig 5A–5D for monkey 1), periodic firing neurons were located slightly more caudal part than non-periodic firing neurons (mean difference = 0.32 mm, p = 0.04, Wilcoxon rank-sum test, Fig 5E for monkey 1). There was also significant difference in the duration of spikes (Fig 5F); the periodic firing neurons exhibited longer spike durations (0.62 ± 0.02 ms, mean ± SEM) than the other neurons (0.55 ± 0.01 ms, p < 0.001, Wilcoxon rank-sum test). Within the several types of periodic firing neurons (as shown in Table 2), we found no significant differences in the recording locations (Fig 5A–5C for monkey 1) or the duration of spikes (Kruskal–Wallis test, *p* >0.05). On the other hand, within the periodic firing neurons, the frequencies of periodic firing were inversely correlated with the duration of spikes, during both the FX- and FV-conditions (r = -0.49 and -0.41 for the FX- and FV-conditions, respectively, Spearman’s rank correlation test, p < 0.001, Fig 5G). The periodic firing neurons that exhibited longer spike durations (>0.8 ms) showed characteristic low frequency periodic firing. Most of these neurons showed periodic firing with 5~16 Hz (N = 22/27, Fig 5G).

## Discussion

We found that a population of PPTN neurons exhibited rhythmic firing by periodically firing single spikes that changed with the behavioral context. Moreover, these periodic firing patterns appeared during highly active states of the neurons. While, the other population of neurons exhibited constant periodic firing throughout the recording epoch. Furthermore, these periodic firing neurons exhibited longer spike durations and were distributed in the slightly caudal part of the PPTN. We speculate that the behavioral context-related rapid changes in rhythmic firing of PPTN neurons might underlie changes in cortical oscillatory activity and regulate the monkey's attentional and vigilance level to perform the task. These dynamics of rhythmic firing of PPTN neurons might be influenced by local property of the PPTN and reverberate large-scale global circuit including the PPTN.

### Rhythmic firing pattern of PPTN neurons

The rhythmic firing properties of PPTN neurons have been reported in various conditions and species, from slice preparations of rats to awake humans [13, 14, 29]. The reported frequencies of regular firing were slightly different among these studies, possibly reflecting several interspecies differences in anatomical connections and physiological properties of the PPTN [30].

In this paper, we found that more than half of rhythmic firing PPTN neurons exhibited low frequency periodic firing (5 to 30 Hz), but others exhibited high frequency firing up to 100 Hz (Fig 4). These neurons showed periodic firing pattern mainly during higher firing rate conditions (Table 2). Previous studies in rat slice preparations reported that PPTN neurons were capable of generating rhythmic activity at gamma band frequency (40–60 Hz) without rhythmic input [29], and suggested that the rhythmic activity appeared to be a part of the intrinsic membrane properties of PPTN neurons [31]. The periodic firing during higher firing rate conditions, here we reported, is consistent with the finding in slice experiment, while the frequency of the periodic firing is more variable possibly reflecting various input condition of *in vivo* experiment.

### Context-dependent changes in periodic firing of PPTN neurons

We previously reported that many PPTN neurons showed a tonic increase or decrease in firing rate related to task execution and reward prediction [16, 17]. Here we showed that a subset of these PPTN neurons changed their firing rhythmicity with changing firing rate (Fig 3). In other words, the periodic firing pattern of PPTN neurons were altered with changing behavioral context that interleaved only a few seconds. This rapid changes of PPTN rhythmic firing might contribute short interval changes in state of attention and/or vigilance, which is consistent with the concept of the PPTN as an ascending reticular activating system [32].

Classically, the ascending reticular activating system including the PPTN is thought to play a role in controlling the sleep/wake state [7–9]. Actually, the firing patterns of single PPTN neurons changes with sleep states [9–11]. Human positron emission tomographic study showed that activity of midbrain tegmentum is higher during high vigilance and general attention state than when participants are awake and resting [33]. Moreover, electrical stimulation of the PPTN evokes a shift from the sleep to the waking state, and leads an increase in gamma oscillations (30–60 Hz) in the cortex [32]. Garcia-rill and his colleagues suggested that rhythmic firing of PPTN neurons would contribute to generation and maintenance of cortical gamma band activity [1]. For lower frequency, single PPTN neuronal firing is phase locked to cortical alpha oscillation [9, 21]. Future experiments are required to examine the relationship between the rhythmic firing of the PPTN and the ongoing global oscillatory activity.

These context-dependent changes in firing rates and firing patterns of PPTN neurons suggest that external inputs play a role in triggering and maintaining the rhythmic firing of PPTN neurons. For the excitatory inputs, the primate PPTN receives direct cortical inputs from multiple motor-related areas of the frontal lobe [34]. Furthermore, rescent study using probabilistic diffusion tractography also showed the functional connections between the frontal motor regions and the PPTN [35]. Of these areas, the supplementary motor area is considered to have a role in anticipatory control of posture, and this process is disturbed in Parkinson’s disease patients [36]. Thus, the excitatory cortical projection to the PPTN might contribute to make profile of firing frequency during the FX-condition (which is shown in Fig 3) as well as higher rhythmic firing capability.

On the other hand, for the inhibitory inputs to the PPTN, there are massive GABAergic inhibitory projections from the basal ganglia output nuclei, including the globus pallidus and substantia nigra pars reticulata [37, 38]. These basal ganglia nuclei exhibited an increase or decrease in firing rate related to behavioral task [39, 40]. Another possible source of the inhibitory input is an oscillatory reverberation between cholinergic PPTN neurons and other serotonergic and noradrenergic neuromodulatory systems, which is classically thought to play a role in alternation and maintenance of rapid eye movement sleep [7, 41]. It has been shown that serotonergic and noradrenergic inputs inhibit the activity of the cholinergic neurons [42, 43].

For non-rhythmically firing PPTN neurons, there were also changes in firing regularity with changing firing rate. The changes in firing regularity reflect the changes in firing rate between the FX- and FV-conditions, such that when the firing rate becomes higher the CV becomes lower (Fig 2C). This relationship in firing rate and CV modulation for the task execution was similar to that of modulation by large/small reward expectation. Thus, changes in firing rate by the task execution and reward expectation might share an identical activation mechanism.

### Neuron type and firing characteristics

The PPTN is a heterogeneous nucleus and contains cholinergic, glutamatergic, and GABAergic neurons [2, 3]. We found that PPTN neurons showed no significant correlation between mean firing rate and CV, possibly reflecting the heterogeneous characteristics of the PPTN (Fig 2A and 2B). The neurotransmitter of the recorded neurons was difficult to determine in our current experiments, however, physiological and anatomical properties may provide a clue to the neurochemical profile of the recorded neurons.

In previous studies, the neurotransmitter of recorded PPTN neurons might be related to their spike durations [23, 24]; though recent study reported that distribution of spike durations from identified cholinergic and non-cholinergic neurons were not different [12]. Anatomically, cholinergic neurons are distributed to the caudal part of the PPTN, while GABAergic neurons are distributed to the rostral part [4, 28].

We found that periodic firing neurons were distributed slightly caudal part of the PPTN (Fig 5E) and exhibited long spike durations (Fig 5F). Especially, neurons that exhibit long spike durations displayed a characteristic periodic firing at 10 Hz (Fig 5G). These characteristics are consistent with the feature of reported cholinergic neurons; however, further studies are needed to identify the relationship between the neurochemical identity and firing pattern.

### Rhythmic firing of the PPTN and Parkinson’s disease

The PPTN is critically related to Parkinson’s disease. Previous studies reported the loss of PPTN cholinergic neurons in Parkinson's disease, and experimental lesions of the PPTN of normal monkeys produced contralateral hemi-parkinsonism [44]. Thus, the degeneration of PPTN neurons or their dysfunction may be important in the pathophysiology of locomotor and postural disturbances of parkinsonism [44].

The PPTN has recently been introduced as a new therapeutic target for deep brain stimulation in Parkinson's patients [45], and effective electrical stimulation in the PPTN is low frequency at 10 Hz. Treatment with levodopa leads to the appearance of alpha band activity (~10 Hz) in the local field potential recorded from the PPTN [13, 14], and pairs of PPTN neurons showed synchronized firing [21]. The rhythmic stimulation might induce periodic activity of PPTN neurons and facilitate network activity that includes the PPTN. This, however, remains to be established.

The single spike rhythmic firing of PPTN neurons, here we reported, should be a basis of the rhythmic population activity. One possible explanation is that the phases of each periodic single unit firing were aligned and might produce oscillatory population response. Although, in our single unit recording study, we have no data about synchronized activity; some previous findings support the oscillatory firing of PPTN neurons, such as the presence of electrically coupled pairs of neurons in the PPTN [46], and the intrinsic cholinergic connections within the PPTN [9]. In future studies, it may be important to investigate the activities of PPTN neurons in the monkey model of Parkinson’s disease. Possibly, the rhythmic population activity of the PPTN might be disrupted by the irregular firing of single neurons or de-coupling of rhythmic activity in population. In Parkinson’s condition, abnormal rhythmic activity were reported in the globus pallidus [47] and subthalamic nucleus [48], both of which exhibit rhythmic firing resulting from repetition of burst activity, that might disrupt the single spike rhythmic firing of PPTN neurons. Elucidating single neuronal mechanisms of the rhythmic population activity would be important to investigate the effects and mechanisms of levodopa and electrical stimulation for treatment of Parkinson’s disease.
